# Melatonin Suppresses Apoptosis of Nucleus Pulposus Cells through Inhibiting Autophagy via the PI3K/Akt Pathway in a High-Glucose Culture

**DOI:** 10.1155/2021/4604258

**Published:** 2021-10-08

**Authors:** Jian Li, Chengqiang Wang, Lixin Xue, Fan Zhang, Jianqiang Liu

**Affiliations:** ^1^Department of Orthopedics the Fourth People's Hospital of Jinan, The Third Affiliated Hospital of Shandong First Medical University, Jinan, China; ^2^Nursing Department the Fourth People's Hospital of Jinan, The Third Affiliated Hospital of Shandong First Medical University, Jinan, China; ^3^Eightieth Group Army Hospital of PLA Army, Weifang, China

## Abstract

Diabetes mellitus- (DM-) associated hyperglycemia promotes apoptosis of disc nucleus pulposus (NP) cells, which is a contributor to intervertebral disc degeneration (IDD). Melatonin is able to protect against cell apoptosis. However, its effects on apoptosis of NP cell in a high-glucose culture remain unclear. The purpose of the present study was to investigate the effects and molecular mechanism of melatonin on NP cell apoptosis in a high-glucose culture. NP cells were cultured in the baseline medium supplemented with a high-glucose concentration (0.2 M) for 3 days. The control cells were only cultured in the baseline medium. Additionally, the pharmaceutical inhibitor LY294002 was added along with the culture medium to investigate the possible role of the PI3K/Akt pathway. Apoptosis, autophagy, and activity of the PI3K/Akt pathway of NP cells among these groups were evaluated. Compared with the control NP cells, high glucose significantly increased cell apoptosis ratio and caspase-3/caspase-9 activity and decreased mRNA expression of Bcl-2, whereas it increased mRNA or protein expression of Bax, caspase-3, cleaved caspase-3, cleaved PARP, and autophagy-related molecules (Atg3, Atg5, Beclin-1, and LC3-II) and decreased protein expression of p-Akt compared with the control cells. Additionally, melatonin partly inhibited the effects of high glucose on those parameters of cell apoptosis, autophagy, and activation of PI3K/Akt. In conclusion, melatonin attenuates apoptosis of NP cells through inhibiting the excessive autophagy via the PI3K/Akt pathway in a high-glucose culture. This study provides new theoretical basis of the protective effects of melatonin against disc degeneration in a DM patient.

## 1. Introduction

Intervertebral disc degeneration- (IDD-) caused low back pain is a painful dilemma around world. It is a main contributor of physical disability and work absence [1]. Recently, several studies demonstrated that diabetes mellitus (DM) is a risk factor of IDD in a DM patient, and DM patients have a higher incidence and a faster progress of IDD than non-DM patients [2–5]. Furthermore, several basic researches reported that DM-associated hyperglycemia is harmful to the health biology of disc cells [6–14]. Currently, many researchers have managed to investigate the pathogenesis of IDD in DM patients.

The individual intervertebral disc (IVD) includes the annulus fibrosus (AF) tissue, nucleus pulposus (NP) tissue, and cartilaginous endplate tissue [15]. With aging and degeneration of IVD, the NP region first exhibits alterations in extracellular matrix biosynthesis and matrix denaturation [16]. In the NP tissue, NP cells are the primary cells which are responsible for matrix biosynthesis and degeneration [17]. Previous studies have reached a consensus that disc NP cell apoptosis plays an implicate role in initiating and accelerating the disc degeneration process [18–25]. Additionally, several studies found that a high-glucose culture facilitates disc cell apoptosis [6, 11, 14, 26]. Hence, attenuation of disc cell apoptosis in the high-glucose environment might be a potential approach to slow down disc degeneration progress in DM patients.

Melatonin is a pineal gland released molecule with low toxicity, high solubility, and important effects on mitochondrial homeostasis [27, 28]. Based on previous studies, melatonin is able to resist oxidative stress reaction, inflammation response, and cellular apoptosis in animal models of cartilage degeneration [29–31]. Cartilage degeneration is a similar pathological progression compared with disc degeneration, and there are few studies that reported the role of melatonin in disc degeneration [32–34]. Previously, several researchers have demonstrated that high glucose promotes cellular apoptosis [7, 11, 14, 35, 36] and accelerates cellular autophagy in disc cells [8, 12]. In light of the excessive autophagy being detrimental to health cellular biology, we deduced that inhibition of excessive autophagy may be able to suppress high glucose-caused apoptosis of NP cells. Therefore, this study was aimed at investigating whether melatonin has protective effects against high-glucose culture-mediated NP cell apoptosis, and the relationship between melatonin, NP cell apoptosis, and autophagy in a high-glucose culture, as well as the potential signaling pathways.

## 2. Materials and Methods

### 2.1. Ethical Statement

The animal experiments of this study are approved by the Ethics Committee at the Third Affiliated Hospital of Shandong First Medical University.

### 2.2. NP Cell Separation and Culture

In total, there are twenty-eight rats (220-250 g weight and 8-9 weeks old) were used for NP cell isolation in the present study. Briefly, after intraperitoneal injection of chloride hydrate with a ratio of 300 mg/kg (dosage/weight), the lumbar spine (L1-L5) was obtained under sterile conditions. Then, the attached soft tissues were removed to obtain intact IVDs, followed by the central NP tissue digestion with 0.25 type II collagenase (Gibco, USA) for 20 minutes in the cell thermostatic incubator. Thereafter, NP cells were resuspended in DMEM/F12 medium supplemented with 15% fetal bovine serum (FBS; Invitrogen, USA) and subcultured when reaching to the 80%-90% confluence. The passage-3 cells were subjected to each assays designed in this study. Here, the control NP cells were just cultured in basic medium whereas the experimental NP cells were cultured in a basic medium supplemented with a high-glucose concentration (0.2 M) for 3 days. Melatonin (1.0 mM, this concentration was referred to a previous study [37]) was used to study its role in regulating apoptosis of NP cells. The pharmaceutical inhibitor LY294002 with a concentration of 10 *μ*M was used to observe the potential effects of the PI3K/Akt pathway.

### 2.3. Flow Cytometry Assay

Briefly, after the collected NP cells floated in the culture medium and attached in the culture plate were rinsed with phosphate buffer solution (PBS) for 3 times, 20 × 10^4^ cells per group were incubated with Annexin V-FITC binding buffer (195 *μ*L), followed by incubation with Annexin V-FITC solution (5 *μ*L) and propidium iodide (PI, 10 *μ*L) solution according to the operation steps (Beyotime, China). Finally, a flow cytometry machine was used to analyze apoptotic cell ratio. Here, both the early and late apoptotic cells were calculated.

### 2.4. Caspase Activity Measurement

In this study, we used specific commercial kit (Beyotime, China) to detect activity of caspase-3 and caspase-9. Briefly, after culture under different conditions, NP cells were collected and treated with lysis buffer provided in the chemical kit. Then, the protein supernatant was used to prepare the reaction system, followed by the measurement of the absorbance value at a wavelength of 405 nm. Based on the prepared standard curve and the absorbance value, activity of caspase-3 and caspase-9 was calculated.

### 2.5. Real-Time Polymerase Chain Reaction (PCR) Analysis

After NP cells were cultured under different conditions, total RNA was extracted from them using TRIzol reagent (Beyotime, China) and 1 *μ*g of RNA was synthesized into cDNA. Then, a Real-Time PCR System (Thermo, USA) was used to perform the real-time PCR with SYBR Green Mix (Qiagen, Germany) for a total of 40 cycles. GAPDH was designed as an internal normalization. Primers of our target genes were showed in Table 1. The relative mRNA expression was calculated according to the method of 2^−△△Ct^.

### 2.6. Western Blot Analysis

After NP cells were cultured under different conditions, they were incubated with RIPA lysis buffer (TIANGEN, Bejing, China) to extract the total protein sample. Thereafter, protein samples (50 *μ*g per group) were separated by 12% sodium salt- (SDS-) polyacrylamide gel electrophoresis (PAGE) gels and transferred to the polyvinylidene fluoride (PVDF) membrane (Millipore, USA). After the PVDF membranes were incubated using 5% bovine serum albumin (BSA, Beyotime, China) to block nonspecific staining sites, they were probed by the specific primary antibodies (GAPDH: Abcam, ab181602; Cleaved caspase-3: Abcam, ab49822; Cleaved PARP: Cell Signaling Technology, #94885; Akt: Cell Signaling Technology, #4685S; Phospho-Akt: Cell Signaling Technology, #4060S; Beclin-1: Abcam, ab207612; and LC3: Cell Signaling Technology, #3868) and the corresponding horse radish peroxidase- (HRP-) conjugated secondary antibodies. Finally, after the protein bands on the PVDF membrane were developed by the enhanced chemiluminescence (ECL) solution, the gray value of target proteins was measured using the Quantity One software (Bio-Rad, USA) and normalized to GAPDH.

### 2.7. Statistical Analysis

All experiment data were represented as means ± standard deviation. The data were analyzed using the SPSS software (version 16.0). Student's *t*-test was performed to measure the statistical difference between two designed groups, whereas the one-way analysis of variance and the following post-LSD test were performed to measure statistical difference among the three designed groups. A statistical difference was suggested when *p* < 0.05.

## 3. Results

### 3.1. Cell Apoptosis Ratio

**Figure 1:**
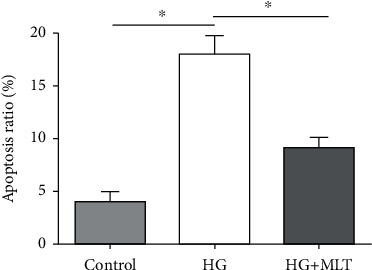
Analysis of nucleus pulposus (NP) cell apoptosis. NP cell apoptosis ratio was detected by flow cytometry. Data are exhibited as mean ± SD, *n* = 3. ^∗^A statistical difference (*p* < 0.05). Con: control; HG: high glucose; HG+MLT(1.0): high glucose+1.0 mM melatonin.

After NP cells were cultured in the medium supplemented with a high-glucose concentration, the NP cell apoptosis ratio was significantly increased compared with the control NP cells. However, addition of melatonin into the high-glucose culture medium partly declined cellular apoptosis ratio (Figure 1).

### 3.2. Caspase-3/Caspase-9 Activity

**Figure 2:**
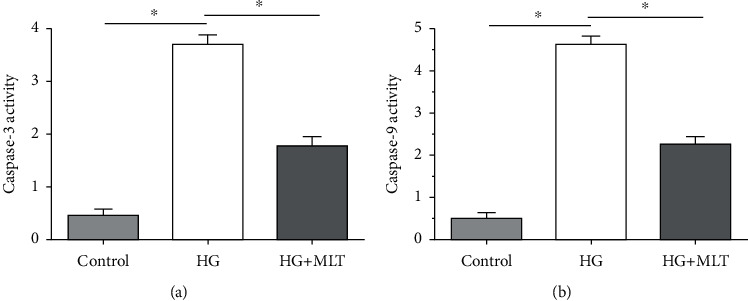
Analysis of caspase-3 and caspase-9 activities in nucleus pulposus (NP) cells. (a, b) Caspase-3 activity and caspase-9 activity, respectively. Data are exhibited as mean ± SD, *n* = 3. ^∗^A statistical difference (*p* < 0.05). Con: control; HG: high glucose; HG+MLT(1.0): high glucose+1.0 mM melatonin.

Results showed that both caspase-3 activity and caspase-9 activity were increased after being cultured in the medium supplemented with a high-glucose concentration. However, melatonin decreased activity of them in a high-glucose environment (Figure 2).

### 3.3. Expression of Apoptosis-Related Molecules

**Figure 3:**
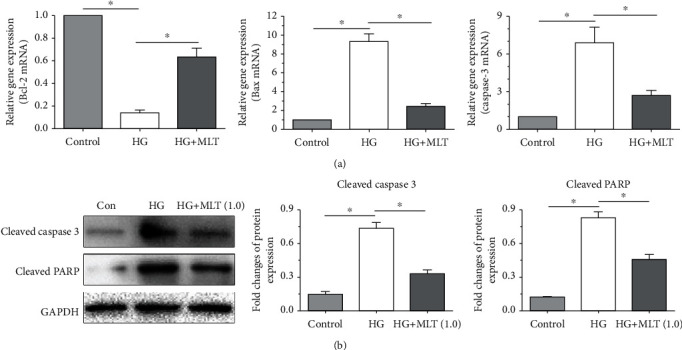
Analysis of mRNA or protein expression of apoptosis-related molecules in nucleus pulposus (NP) cells. (a, b) The mRNA expression results and protein expression results, respectively. Data are exhibited as mean ± SD, *n* = 3. ^∗^A statistical difference (*p* < 0.05). Con: control; HG: high glucose; HG+MLT(1.0): high glucose+1.0 mM melatonin.

In this study, the mRNA expression of several common proapoptosis genes (Bax and caspase-3) and antiapoptosis gene (Bcl-2) was measured. NP cells cultured in the medium supplemented with a high-glucose concentration showed an increase in the mRNA expression of Bax and caspase-3 and a decrease in the mRNA expression of Bcl-2 compared with the control NP cells. Results also showed that addition of melatonin in the high-glucose group partly decreased mRNA expression of Bax and caspase-3 and increased mRNA expression of Bcl-2 (Figure 3(a)). To further investigate cell apoptosis, the protein expression of two classical apoptosis proteins (cleaved caspase-3 and cleaved PARP) was also evaluated in this study. Results showed that NP cells cultured in the medium supplemented with a high-glucose concentration exhibited an increased protein expression of both of these two apoptosis markers compared with the control NP cells, whereas addition of melatonin downregulated their expression level in NP cells cultured in the medium supplemented with a high-glucose concentration (Figure 3(b)).

### 3.4. Expression of Autophagy-Related Molecules

**Figure 4:**
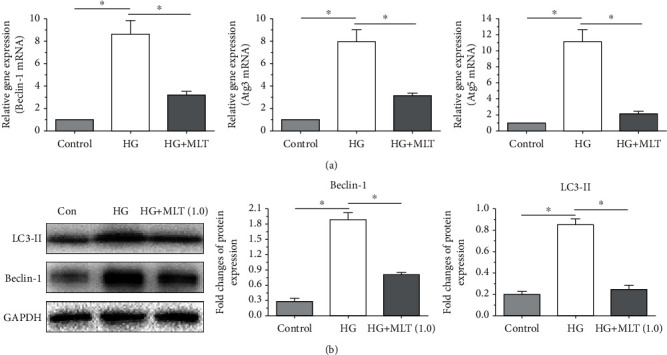
Analysis of mRNA or protein expression of autophagy-related molecules in nucleus pulposus (NP) cells. (a, b) The mRNA expression results and protein expression results, respectively. Data are exhibited as mean ± SD, *n* = 3. ^∗^A statistical difference (*p* < 0.05). Con: control; HG: high glucose; HG+MLT(1.0): high glucose+1.0 mM melatonin.

In this study, mRNA expression of several autophagy-related genes (Beclin-1, Atg3, and Atg5) was detected. Results demonstrated that a high-glucose culture obviously increased mRNA expression of Beclin-1, Atg3, and Atg5 compared with the control cells. On the contrary, addition of melatonin partly decreased mRNA expression of them in NP cells cultured in the medium supplemented with a high-glucose concentration (Figure 4(a)). Additionally, the protein expression of autophagy molecules (Beclin-1 and LC3) was also evaluated here. The result demonstrated that a high-glucose culture significantly elevated protein expression of Beclin-1 and LC3-II compared with the control cells. However, melatonin partly decreased the protein expression of Beclin-1 and LC3-II in NP cells cultured in the medium supplemented with a high-glucose concentration (Figure 4(b)).

### 3.5. Activity of the PI3K/Akt Signaling Pathway

**Figure 5:**
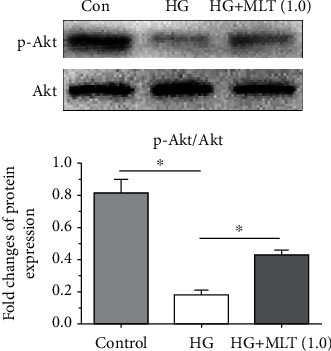
Analysis of activity of the PI3K/Akt pathway. Its activity was indicated by expression of p-Akt that was measured by western blot assay. Data are exhibited as mean ± SD, *n* = 3. ^∗^A statistical difference (*p* < 0.05). Con: control; HG: high glucose; HG+MLT(1.0): high glucose+1.0 mM melatonin.

In order to investigate whether the PI3K/Akt signaling pathway functioned in this regulatory process, we detected protein expression of p-Akt to reflect the activity of the PI3K/Akt signaling pathway. Results demonstrated that a high-glucose culture obviously decreased the protein expression of p-Akt compared with the control NP cells, but addition of melatonin partly promoted protein expression of p-Akt in NP cells cultured in the medium supplemented with a high-glucose concentration (Figure 5).

### 3.6. Effects of PI3K/Akt Pathway Inhibition on NP Cell Apoptosis

**Figure 6:**
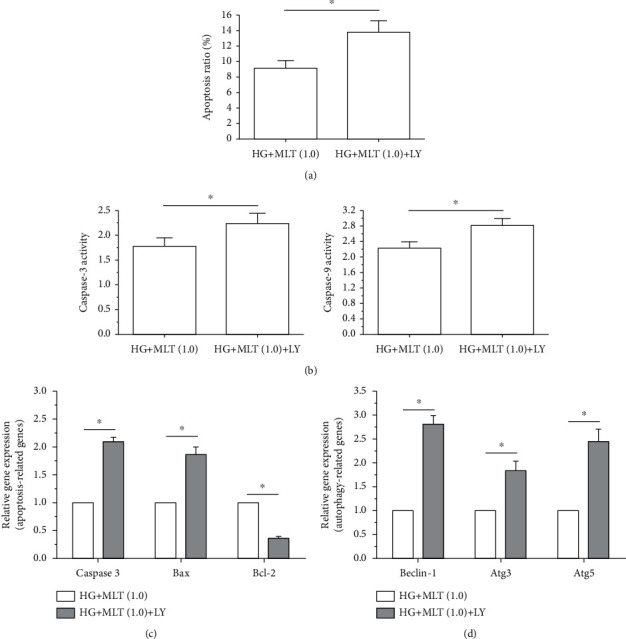
Inhibition of the PI3K/Akt pathway attenuated nucleus pulposus (NP) cell apoptosis in a high-glucose culture. (a–d) Results of cell apoptosis ratio, caspase-3/caspase-9 activity, mRNA expression of apoptosis-related molecules, and mRNA expression of autophagy-related molecules, respectively. Data are exhibited as mean ± SD, *n* = 3. ^∗^A statistical difference (*p* < 0.05). Con: control; HG: high glucose; HG+MLT(1.0): high gl ucose+1.0 mM melatonin.

To verify the role of the PI3K/Akt signaling pathway in this regulatory process, the inhibitor LY294002 was used to inhibit activation of this signaling pathway in melatonin (1.0 mM)-treated NP cells in the high-glucose culture. Results revealed that when the inhibitor LY294002 inhibited activation of the PI3K/Akt pathway in melatonin (1.0 mM)-treated NP cells under a high-glucose culture, they exhibited a significant decrease in the cell apoptosis ratio (Figure 6(a)) and caspase-3/caspase-9 activity (Figure 6(b)), an obvious increase in mRNA expression of proapoptosis genes (Bax and caspase-3) but a decrease in mRNA expression of the antiapoptosis gene (Bcl-2) (Figure 6(c)), and a significant upregulation in the mRNA expression of autophagy-associated genes (Beclin-1, Atg3, and Atg5) (Figure 6(d)).

## 4. Discussion

IDD is a topic in the field of spine research. It has been well established that disc degeneration-related disease is a main reason for low back pain [1]. Recent studies have demonstrated that DM-associated hyperglycemia is a contributor to disc degeneration in DM patients [2–4]. High glucose is proved to promote disc cell apoptosis which is an important classical process during disc degeneration [7, 11, 14]. Melatonin is effective in resisting oxidative stress reaction, inflammation response, and cellular apoptosis in animal models of cartilage degeneration [29–31]. The present study was mainly aimed at investigating the role and the molecular mechanism of melatonin in regulating high glucose-induced NP cell apoptosis.

Hyperglycemia is an implicate microenvironment in DM patients. Several previous studies have well studied the role of high glucose in mediating disc cell biology and found that a high-glucose culture obviously promoted apoptosis of disc NP cells [7, 11, 14]. In our study, we found that a high-glucose culture obviously elevated NP cell apoptosis ratio and caspase-3/caspase-9 activity, decreased the mRNA expression of Bcl-2, and increased the mRNA or protein expression of some proapoptosis molecules (Bax, caspase 3, cleaved caspase-3, and cleaved PARP) compared with the control cells, indicating that a high-glucose microenvironment aggravates disc NP cell apoptosis in this study. Our results confirm and are in line with previous studies [7, 11, 14].

Autophagy is a physiological event and participates in many cell biological behaviors [38]. Appropriate cellular autophagy is helpful to maintain the cellular homeostasis but excessive autophagy has detrimental effects on cell biology [39, 40]. Several previous studies have indicated that a high-glucose environment promotes cellular autophagy of NP cells [8] and mediates oxidative stress injury through enhancing autophagy in disc notochordal cells [12]. In this study, we found that a high-glucose culture significantly increased the mRNA expression of Beclin-1, Atg3, and Atg5 and protein expression of Beclin-1 and LC3-II compared with the control cells, indicating that a high-glucose culture exacerbates cellular autophagy in disc NP cells. These results are consistent with aforementioned previous studies.

Melatonin is a pineal gland released molecule with low toxicity, high solubility, and important effects on mitochondrial homeostasis [27, 28]. Several research teams have reported the effects of melatonin on biological behaviors in disc cells. Chen et al. have reported that melatonin ameliorates disc degeneration through inhibition cell apoptosis [32]; Zhang et al. have found that melatonin protects cartilage endplate cell apoptosis via the SIRT1-autophagy pathway [34]; He et al. have showed that melatonin resists oxidative stress-induced disc NP cell apoptosis [33]. These studies showed that there is a close relationship among melatonin, autophagy, and cell apoptosis. Here, our results showed that melatonin decreased the apoptosis ratio and caspase-3/caspase-9 activity and increased the mRNA expression of Bcl-2, whereas it decreased the mRNA or protein expression of Bax, caspase 3, cleaved caspase-3, and cleaved PARP in high glucose-cultured disc NP cells, indicating that melatonin can attenuate high glucose-induced apoptosis of disc NP cells. On another hand, the results demonstrated that melatonin decreased the mRNA expression of Beclin-1, Atg3, and Atg5 and protein expression of Beclin-1 and LC3-II in high glucose-cultured disc NP cells, indicating that melatonin suppresses high glucose-induced excessive cellular autophagy. Because excessive autophagy has detrimental effects on cell biology [39, 40], we can deduce that melatonin may suppress high glucose-caused NP cell apoptosis via suppressing the excessive cell autophagy.

The PI3K/Akt signaling pathway is a common pathway that is involved in many cellular biological behaviors of disc cells, such as proliferation, senescence, apoptosis and cellular biosynthesis [14, 24, 41–45]. Furthermore, melatonin can activate the PI3K/Akt pathway to meditate its protective effects in other cell types [46–48]. In the present study, we showed that high glucose obviously decreased the protein expression of p-Akt whereas melatonin partly increased the protein expression of p-Akt in the high glucose-cultured NP cells, indicating that melatonin can resist high-glucose culture-induced inhibition of the PI3K/Akt pathway. Based on the results of cell apoptosis and cellular autophagy described above, we further speculate that melatonin suppresses high glucose-caused NP cell apoptosis through inhibiting excessive autophagy via the PI3K/Akt pathway.

This study has several shortcomings. First, because the rat NP tissue contains some notochordal cells, the separated NP cell pellets may contain some notochordal cells. Here, we are not sure that the existence of them may cause how much influence on our results. Second, the present study is an absolute in vitro study. If an additional in vivo study was performed, our conclusion may even be more persuasive.

## 5. Conclusion

Together, this study investigated the role and the potential molecular mechanism of melatonin in regulating NP cell apoptosis in a high-glucose culture. Our results demonstrated that melatonin can inhibit disc NP cell apoptosis through suppressing the excessive autophagy in a high-glucose culture, and the PI3K/Akt signaling pathway may be responsible for the protective effects of melatonin against high-glucose culture-caused NP cell apoptosis. This study proves the theoretical basis of the protective effects of melatonin against disc degeneration in DM patients and is helpful to further understand the potential molecular mechanism behind this regulation.

## Figures and Tables

**Table 1:** Primers of target genes.

Gene	Forward (5′-3′)	Reverse (5′-3′)
GAPDH	CCGCGAGTACAACCTTCTTG	TGACCCATACCCACCATCAC
Bcl-2	GGGGCTACGAGTGGGATACT	GACGGTAGCGACGAGAGAAG
Bax	CCAGGACGCATCCACCAAGAAG	GCTGCCACACGGAAGAAGACC
Caspase-3	GTACAGAGCTGGACTGCGGTATTG	AGTCGGCCTCCACTGGTATCTTC
Beclin-1	AGGAGTTGCCGTTGTACTGTTCTG	TGCCTCCAGTGTCTTCAATCTTGC
Atg3	TGGAAGTGGCCGAGTACCTGAC	GCCATGTTGGACAGTGGTGGAC
Atg5	CTCAGCTCTGCCTTGGAACATCAC	AAGTGAGCCTCAACTGCATCCTTG

## Data Availability

All data were included in the text.

## References

[1] Macfarlane G. J., Thomas E., Croft P. R., Papageorgiou A. C., Jayson M. I., Silman A. J. (1999). Predictors of early improvement in low back pain amongst consulters to general practice: the influence of pre-morbid and episode-related factors. *Pain*.

[2] Fabiane S. M., Ward K. J., Iatridis J. C., Williams F. M. (2016). Does type 2 diabetes mellitus promote intervertebral disc degeneration?. *European Spine Journal*.

[3] Liu X., Pan F., Ba Z., Wang S., Wu D. (2018). The potential effect of type 2 diabetes mellitus on lumbar disc degeneration: a retrospective single-center study. *Journal of Orthopaedic Surgery and Research*.

[4] Jakoi A. M., Pannu G., D'Oro A. (2017). The clinical correlations between diabetes, cigarette smoking and obesity on intervertebral degenerative disc disease of the lumbar spine. *Asian Spine Journal.*.

[5] Anekstein Y., Smorgick Y., Lotan R. (2010). Diabetes mellitus as a risk factor for the development of lumbar spinal stenosis. *The Israel Medical Association Journal*.

[6] Guo M. B., Wang D. C., Liu H. F. (2018). Lupeol against high-glucose-induced apoptosis via enhancing the anti-oxidative stress in rabbit nucleus pulposus cells. *European Spine Journal*.

[7] Jiang Z., Lu W., Zeng Q., Li D., Ding L., Wu J. (2018). High glucose-induced excessive reactive oxygen species promote apoptosis through mitochondrial damage in rat cartilage endplate cells. *Journal of Orthopaedic Research*.

[8] Kong C. G., Park J. B., Kim M. S., Park E. Y. (2014). High glucose accelerates autophagy in adult rat intervertebral disc cells. *Asian Spine Journal.*.

[9] Kong J. G., Park J. B., Lee D., Park E. Y. (2015). Effect of high glucose on stress-induced senescence of nucleus pulposus cells of adult rats. *Asian Spine Journal.*.

[10] Li P., Gan Y., Wang H. (2017). A substance exchanger-based bioreactor culture of pig discs for studying the immature nucleus pulposus. *Artificial Organs*.

[11] Liu Z., Zhang Z., Zhang A. (2019). Osteogenic protein-1 alleviates high glucose microenvironment-caused degenerative changes in nucleus pulposus cells. *Bioscience Reports*.

[12] Park E. Y., Park J. B. (2013). High glucose-induced oxidative stress promotes autophagy through mitochondrial damage in rat notochordal cells. *International Orthopaedics*.

[13] Park J. S., Park J. B., Park I. J., Park E. Y. (2014). Accelerated premature stress-induced senescence of young annulus fibrosus cells of rats by high glucose-induced oxidative stress. *International Orthopaedics*.

[14] Wang W., Li P., Xu J. (2018). Resveratrol attenuates high glucose-induced nucleus pulposus cell apoptosis and senescence through activating the ROS-mediated PI3K/Akt pathway. *Bioscience Reports*.

[15] Schollum M. L., Robertson P. A., Broom N. D. (2009). A microstructural investigation of intervertebral disc lamellar connectivity: detailed analysis of the translamellar bridges. *Journal of Anatomy*.

[16] Antoniou J., Steffen T., Nelson F. (1996). The human lumbar intervertebral disc: evidence for changes in the biosynthesis and denaturation of the extracellular matrix with growth, maturation, ageing, and degeneration. *The Journal of Clinical Investigation.*.

[17] Lotz J. C., Staples A., Walsh A., Hsieh A. H. (2004). Mechanobiology in intervertebral disc degeneration and regeneration. *Conference Proceedings*.

[18] Ding F., Shao Z. W., Xiong L. M. (2013). Cell death in intervertebral disc degeneration. *Apoptosis*.

[19] Galanti C., Musumeci G., Valentino J., Giunta S., Castorina S. (2013). A role for apoptosis in temporomandibularjoint disc degeneration. A contemporary review. *Italian Journal of Anatomy and Embryology*.

[20] Zhang F., Zhao X., Shen H., Zhang C. (2016). Molecular mechanisms of cell death in intervertebral disc degeneration (review). *International Journal of Molecular Medicine*.

[21] Zhao C. Q., Zhang Y. H., Jiang S. D., Jiang L. S., Dai L. Y. (2010). Both endoplasmic reticulum and mitochondria are involved in disc cell apoptosis and intervertebral disc degeneration in rats. *Age*.

[22] Li P., Gan Y., Wang H. (2017). Role of the ERK1/2 pathway in osmolarity effects on nucleus pulposus cell apoptosis in a disc perfusion culture. *Journal of Orthopaedic Research*.

[23] Li P., Gan Y., Wang H. (2016). Dynamic compression effects on immature nucleus pulposus: a study using a novel intelligent and mechanically active bioreactor. *International Journal of Medical Sciences*.

[24] Li P., Liang Z., Hou G. (2018). N-cadherin-mediated activation of PI3K/Akt-GSK-3*β* signaling attenuates nucleus pulposus cell apoptosis under high-magnitude compression. *Cellular Physiology and Biochemistry*.

[25] Jiao S., Li J., Liu B., Yang M., Xiu J., Qu D. (2018). Nucleus pulposus cell apoptosis is attenuated by CDMP-2 through regulating oxidative damage under the hyperosmotic environment. *Bioscience Reports*.

[26] Qi L., Wang R., Shi Q., Yuan M., Jin M., Li D. (2019). Umbilical cord mesenchymal stem cell conditioned medium restored the expression of collagen II and aggrecan in nucleus pulposus mesenchymal stem cells exposed to high glucose. *Journal of Bone and Mineral Metabolism*.

[27] García J. J., López-Pingarrón L., Almeida-Souza P. (2014). Protective effects of melatonin in reducing oxidative stress and in preserving the fluidity of biological membranes: a review. *Journal of Pineal Research*.

[28] Leon J., Acuna-Castroviejo D., Escames G., Tan D. X., Reiter R. J. (2005). Melatonin mitigates mitochondrial malfunction. *Journal of Pineal Research*.

[29] Pei M., He F., Wei L., Rawson A. (2009). Melatonin enhances cartilage matrix synthesis by porcine articular chondrocytes. *Journal of Pineal Research*.

[30] Lim H. D., Kim Y. S., Ko S. H. (2012). Cytoprotective and anti-inflammatory effects of melatonin in hydrogen peroxide-stimulated CHON-001 human chondrocyte cell line and rabbit model of osteoarthritis via the SIRT1 pathway. *Journal of Pineal Research*.

[31] Liu X., Gong Y., Xiong K. (2013). Melatonin mediates protective effects on inflammatory response induced by interleukin-1 beta in human mesenchymal stem cells. *Journal of Pineal Research*.

[32] Chen Y., Wu Y., Shi H. (2019). Melatonin ameliorates intervertebral disc degeneration via the potential mechanisms of mitophagy induction and apoptosis inhibition. *Journal of Cellular and Molecular Medicine*.

[33] He R., Cui M., Lin H. (2018). Melatonin resists oxidative stress-induced apoptosis in nucleus pulposus cells. *Life Sciences*.

[34] Zhang Z., Lin J., Tian N. (2019). Melatonin protects vertebral endplate chondrocytes against apoptosis and calcification via the Sirt1-autophagy pathway. *Journal of Cellular and Molecular Medicine*.

[35] Park E. Y., Park J. B. (2013). Dose- and time-dependent effect of high glucose concentration on viability of notochordal cells and expression of matrix degrading and fibrotic enzymes. *International Orthopaedics*.

[36] Zhang C. X., Wang T., Ma J. F., Liu Y., Zhou Z. G., Wang D. C. (2017). Protective effect of CDDO-ethyl amide against high-glucose-induced oxidative injury via the Nrf2/HO-1 pathway. *The Spine Journal*.

[37] Chen F., Jiang G., Liu H. (2020). Melatonin alleviates intervertebral disc degeneration by disrupting the IL-1*β*/NF-*κ*B-NLRP3 inflammasome positive feedback loop. *Bone Research*.

[38] Srinivas V., Bohensky J., Zahm A. M., Shapiro I. M. (2009). Autophagy in mineralizing tissues: microenvironmental perspectives. *Cell Cycle*.

[39] Wang S. Y., Yu Q. J., Zhang R. D., Liu B. (2011). Core signaling pathways of survival/death in autophagy-related cancer networks. *The International Journal of Biochemistry & Cell Biology.*.

[40] Chen Z. F., Li Y. B., Han J. Y. (2011). The double-edged effect of autophagy in pancreatic beta cells and diabetes. *Autophagy*.

[41] Gao J., Zhang Q., Song L. (2018). Resveratrol enhances matrix biosynthesis of nucleus pulposus cells through activating autophagy via the PI3K/Akt pathway under oxidative damage. *Bioscience Reports*.

[42] Gong C., Pan W., Hu W., Chen L. (2019). Bone morphogenetic protein-7 retards cell subculture-induced senescence of human nucleus pulposus cells through activating the PI3K/Akt pathway. *Bioscience Reports*.

[43] Jiang Y., Xie Z., Yu J., Fu L. (2019). Resveratrol inhibits IL-1*β*-mediated nucleus pulposus cell apoptosis through regulating the PI3K/Akt pathway. *Bioscience Reports*.

[44] Ouyang Z. H., Wang W. J., Yan Y. G., Wang B., Lv G. H. (2017). The PI3K/Akt pathway: a critical player in intervertebral disc degeneration. *Oncotarget*.

[45] Yang Y., Wang X., Liu Z., Xiao X., Hu W., Sun Z. (2018). Osteogenic protein-1 attenuates nucleus pulposus cell apoptosis through activating the PI3K/Akt/mTOR pathway in a hyperosmotic culture. *Bioscience Reports*.

[46] Song C., Zhao J., Fu B. (2017). Melatonin-mediated upregulation of Sirt3 attenuates sodium fluoride-induced hepatotoxicity by activating the MT1-PI3K/AKT-PGC-1*α* signaling pathway. *Free Radical Biology & Medicine*.

[47] Wang X., Meng K., He Y., Wang H., Zhang Y., Quan F. (2019). Melatonin stimulates STAR expression and progesterone production via activation of the PI3K/AKT pathway in bovine theca cells. *International Journal of Biological Sciences*.

[48] Zhang Y., Wei Z., Liu W. (2017). Melatonin protects against arsenic trioxide-induced liver injury by the upregulation of Nrf2 expression through the activation of PI3K/AKT pathway. *Oncotarget*.

